# Combining age, sex, body mass index, sport level, and preoperative quadriceps strength improves the predictive ability of quadriceps strength recovery after anterior cruciate ligament reconstruction

**DOI:** 10.1007/s00167-023-07492-y

**Published:** 2023-06-24

**Authors:** Yuya Ueda, Takehiko Matsushita, Yohei Shibata, Kohei Takiguchi, Kumiko Ono, Akihiro Kida, Kyohei Nishida, Kanto Nagai, Yuichi Hoshino, Tomoyuki Matsumoto, Yoshitada Sakai, Ryosuke Kuroda

**Affiliations:** 1 Kobe University Graduate School of Health Sciences, Kobe, Japan; 2 Division of Rehabilitation Medicine, Kobe University Hospital, Kobe, Japan; 3 Department of Orthopaedic Surgery, Kobe University Graduate School of Medicine, 7‑5‑2, Kusunoki‑cho, Chuo‑ku, Kobe, Hyogo 650‑0017 Japan; 4 Division of Rehabilitation Medicine, Kobe University Graduate School of Medicine, Kobe, Japan

**Keywords:** Quadriceps strength, Anterior cruciate ligament, Hamstring autograft, Knee function

## Abstract

**Purpose:**

This study compared the predictive ability of each independent predictor with that of a combination of predictors for quadriceps strength recovery one year after anterior cruciate ligament (ACL) reconstruction.

**Methods:**

Patients who underwent primary ACL reconstruction using hamstring autografts were enrolled. Quadriceps strength, hamstring strength, and anterior tibial translation were measured, and the limb symmetry index (LSI) of the quadriceps and the hamstrings was calculated preoperatively and one year after surgery. Patients were classified into two groups according to the LSI of the quadriceps strength at one year postoperatively (≥ 80% or < 80%). Multivariate logistic regression analysis identified the independent predictors of quadriceps strength recovery, and the cut-off value was calculated using the receiver operating characteristic curve. A model assessing predictive ability of the combination of independent predictors was created, and the area under the curve (AUC) for each independent predictor was calculated by using the receiver-operating characteristic curves and the DeLong method.

**Results:**

Of the 646 patients, 414 (64.1%) had an LSI of at least 80% for quadriceps strength one year after surgery, and 232 patients (35.9%) had an LSI of < 80%. Age, sex, body mass index (BMI), preinjury sport level, and LSI of preoperative quadriceps strength were independently associated with quadriceps strength recovery one year after ACL reconstruction. The cut-off values were age: 22.5 years; sex: female; BMI: 24.3 kg/m^2^; preinjury sport level: no sport; and LSI of preoperative quadriceps strength: 63.3%. The AUC of the model assessing the predictive ability of the combination of age, sex, BMI, preinjury sport level, and LSI of preoperative quadriceps strength was significantly higher (0.73) than that of similar factors of preoperative quadriceps strength (AUC: 0.63, 0.53, 0.56, 0.61, and 0.68, *p* < 0.01, respectively).

**Conclusion:**

The combination of age, sex, BMI, preinjury sport level, and LSI of preoperative quadriceps strength had a superior predictive ability for quadriceps strength recovery at one year after ACL reconstruction than these predictors alone. Multiple factors, including patient characteristics and preoperative quadriceps strength, should be considered when planning rehabilitation programs to improve quadriceps strength recovery after ACL reconstruction.

**Level of evidence:**

III.

## Introduction

Anterior cruciate ligament (ACL) reconstruction improves knee laxity [20, 22]; however, previous studies have reported that quadriceps muscles were still weak after surgery [5, 18]. Quadriceps strength recovery after ACL reconstruction was associated with postoperative hop performance [4, 6, 34], self-reported knee function [6, 14, 36], patellar cartilage injury [10, 31], and return to preinjury sports [1, 17, 21]. Therefore, preoperative identification of patients predicted to have poor quadriceps strength recovery is essential for creating an individualized rehabilitation program.

Factors that affect poor quadriceps strength recovery reportedly include older age [13, 16, 29], female sex [9, 19, 29], high body mass index (BMI) [11], low preinjury sports level [25, 26], delayed ACL reconstruction [32], use of a bone-patellar tendon bone (BTB) graft [27, 33], concomitant cartilage injury [8], and low preoperative quadriceps strength [7, 24, 30]. While there have been many reports on these relationships, only a few studies have examined the predictive ability of these factors. One study reported the predictive ability of preoperative quadriceps strength for quadriceps strength recovery at six months after ACL reconstruction. However, the predictive ability of this single factor was low (sensitivity, 69.1%; specificity, 61.5%; area under the curve (AUC), 0.65) [29], suggesting that the predictive ability of independent predictors alone may be low due to the complex relationship between various factors. Shibata et al. [25] constructed a prediction model by performing a decision tree analysis of age, preinjury Tegner activity scale score, and preoperative quadriceps strength to predict quadriceps strength recovery after ACL reconstruction. Although the report showed that 46.8% of the patients were correctly categorized into groups, it remains unclear whether the predictive ability of the combination of independent predictors is better than that of the independent predictors alone. Constructing a prediction model with superior predictive ability will help to plan better rehabilitation programs and improve the outcomes of patients after ACL reconstruction.

The aim of this study was to examine and compare the predictive abilities of independent predictors alone and the combination of independent predictors for quadriceps strength recovery one year after ACL reconstruction. The hypothesis was that a combination of independent predictors could better predict quadriceps strength recovery one year after ACL reconstruction than independent predictors alone.

## Materials and methods

This study was approved by the institutional review board and the ethics committee of our institution (Approval No. B190055). The study was performed in accordance with the ethical standards put forward in the 1964 Declaration of Helsinki and its later amendments or comparable ethical standards. Informed consent was obtained from all the patients in this study.

This retrospective study was conducted at a single center. The inclusion criteria were as follows: (1) patients who underwent primary unilateral ACL reconstruction between 2003 and 2021 and (2) patients who underwent knee functional measurements both preoperatively (within one month before surgery) and approximately one year (12 ± 2 months) after ACL reconstruction. The exclusion criteria were as follows: (1) patients who underwent bilateral ACL reconstruction, (2) patients who underwent multiligament reconstruction, (3) patients who received additional treatment such as mosaicplasty, knee osteotomy, or ganglion resection except for meniscus repair or meniscectomy, and (4) patients with a history of ACL reconstruction on the ipsilateral or contralateral side. Additionally, (5) patients who underwent orthopaedic knee surgery before ACL reconstruction, (6) patients who underwent ACL reconstruction using a BTB graft, (7) patients who sustained a second ACL injury (graft reruptures or contralateral tears) within one year after ACL surgery, and (8) patients with missing data were excluded.

### Patient demographics and surgical data

The following demographic data were obtained from patient interviews and patient medical records: age at the time of surgery, sex, BMI (weight/(height/100)^2^), preinjury sports activity level (athlete, recreation, and no sport), and waiting period (time from injury to surgery). All patients underwent hamstring tendon (semitendinosus and/or gracilis) autografting, including single-bundle (SB) or double-bundle (DB) ACL reconstructions. Surgical data, including surgical technique (SB or DB), medial or lateral meniscus injury requiring surgical treatment (repair or meniscectomy), and cartilage injury, were collected.

### Rehabilitation

All patients underwent the same postoperative time-based rehabilitation protocol for the first six months. This protocol focused on improving range of motion and knee muscle strength and relieving functional limitations. Cryotherapy, electrostimulation, progressive range-of-motion (ROM) training, and partial weight-bearing with crutches were initiated the day after surgery if the patient could tolerate the treatments. Full weight bearing with a knee brace was allowed beginning two weeks postoperatively. Weight bearing and ROM exercises were delayed for one or two weeks if concomitant meniscus repair was performed for incomplete or complete tears. After discharge, rehabilitation was provided at outpatient rehabilitation centers in hospitals or clinics. Closed kinetic chain exercises such as squats, sidesteps, and cycling on an ergometer were started two weeks postoperatively, and open kinetic chain exercises were started eight weeks postoperatively. Jogging was allowed approximately three months postoperatively. Between three and six months after surgery, muscle strengthening training with progressively increasing exercise load and ROM exercises to achieve full ROM were performed. Neuromuscular training for improving dynamic stability, such as jumps, turning, and cutting, was initiated during this time. Sport-specific exercises were started six months after surgery, and the optimal time to return to preinjury sports was decided by the surgeon approximately nine months postoperatively based on the patient’s knee condition, including swelling and ROM, and the patient’s ability to successfully perform sport-specific exercises. The patients who met the following criteria were allowed to return to sports: an LSI of isokinetic at 60°/s quadriceps and hamstring strength ≥ 90%, an LSI of shingle-leg hop performance ≥ 90%, and no major problems during sport-specific movements. The surgeon, therapist, trainer, coach, and patient collaboratively decided the optimal time to return to sports.

### Knee functional test measurements

Isokinetic muscle strength of the quadriceps and hamstrings at 60°/s was measured both preoperatively and one year after the surgery using two isokinetic dynamometers (MYORET RZ-450; Kawasaki Heavy Industries, Ltd. Hyogo, Japan; and Genu PLUS: Inter Reha Co., Ltd., Tokyo, Japan). MYORET RZ-450 was used from 2003 to 2016, and Genu PLUS was used from 2017 to the present. This muscle strength test was performed after a 5-min warm-up at low resistance using a stationary cycling ergometer. The muscle strength test consisted of two practice contractions and five maximal effort contractions with the healthy limb first followed by the operated limb. Peak torques in extension and flexion were recorded, and the limb symmetry index (LSI) was calculated using the following equation: the peak torque of the operated leg was normalized by the nonoperated leg and multiplied by 100. The reliability of the knee strength measurements was confirmed by performing knee strength measurements in 12 knees of 6 healthy male volunteers. The tests were performed at more than three days but less than seven days after the operation. The agreements between the two measurements were evaluated using the intraclass correlation coefficient (ICC) [28]. The ICCs (1, 1) for isokinetic quadriceps and hamstring strength at 60°/s were 0.85 and 0.70, respectively. These results indicated excellent/good test–retest reliability between the two dynamometer measurements.

Anterior tibial translation at manual maximum force was measured using a KT-2000 arthrometer, and the side-to-side difference in anteroposterior tibial displacement between the operated and nonoperated knees was recorded.

### Outcome

In Burgi et al.’s study [2], the LSI of quadriceps strength was 80%, which was reported as the lower criterion for returning to sports. Therefore, in this study, the patients were divided into two groups: the recovery and the nonrecovery groups, according to an LSI of quadriceps strength of 80% or more and less than 80% at one year after surgery.

### Statistical analysis

Characteristic data, surgical data, and preoperative knee function were compared between the recovery and nonrecovery groups using unpaired *t* tests, Mann–Whitney *U* tests, and *χ*^2^ analyses. A multivariate logistic regression analysis with the forced entry of all variables was performed to identify the independent predictors of quadriceps strength recovery.

To examine the predictive ability of the combination of independent predictors of quadriceps strength recovery one year after ACL reconstruction, a model assessing the predictive ability of the combination of independent predictors of quadriceps strength recovery that were identified in the multivariate logistic regression analysis was created. The AUCs were calculated from the receiver operating characteristic (ROC) curve analysis using the Youden index [35] and were compared using the DeLong method. Net reclassification improvement (NRI) and integrated discrimination improvement (IDI) were calculated to assess whether combining the independent predictors could better predict quadriceps strength recovery [3, 23].

The patients were divided into groups according to the cut-off value of each predictor. The proportion of patients in the recovery and nonrecovery groups was calculated according to the number of cut-off value matches. The Jonckheere–Terpstra trend test was performed to determine whether the nonrecovery group had a higher number of patients who met the criteria. Statistical analyses were performed using R for Windows (version 4.0.2). Statistical significance was determined if the p value was less than 0.05. Considering the number of independent variables in the multiple logistic regression analysis, the minimum sample size was 130 patients for both the recovery and nonrecovery groups.

## Results

**Fig. 1:**
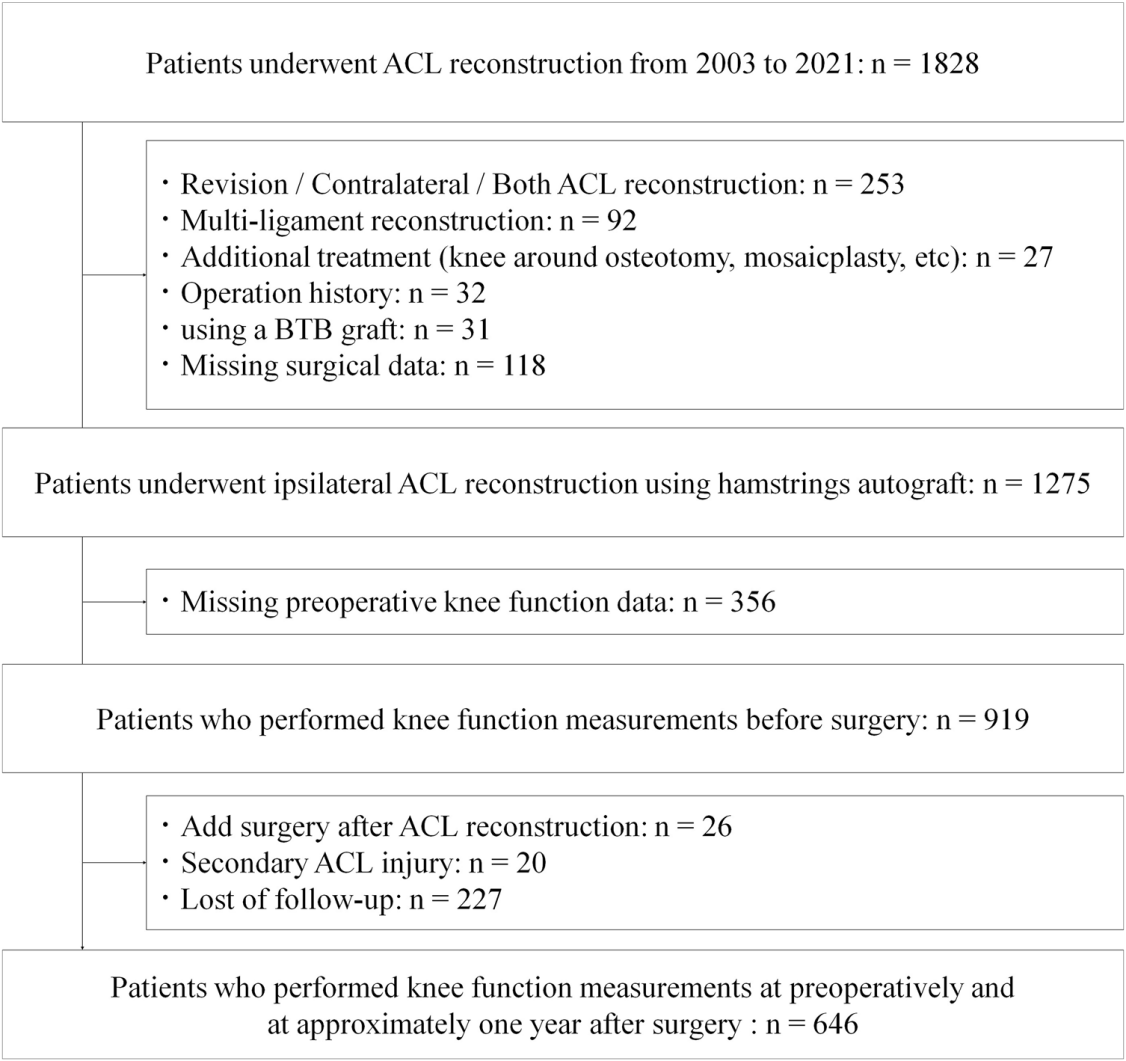
Flowchart of patient inclusion. *ACL* anterior cruciate ligament, *BTB* bone-patellar tendon-bone

This study included 646 patients (Fig. 1). Of the 646 eligible patients, 414 (64.1%) were included in the recovery group, and 232 (35.9%) were included in the nonrecovery group. Four hundred sixty-two patients underwent preoperative knee function tests within two weeks before surgery. There was a weak positive correlation between the waiting time and the preoperative LSI of quadriceps strength (*r* = 0.19, *p* < 0.001) and a weak negative correlation between the waiting time and the LSI of quadriceps strength at one year postoperatively (*r* = − 0.11, *p* = 0.005). Univariate analysis revealed that patients in the recovery group were significantly younger and had a lower BMI, a higher preinjury sport level, and a higher LSI of preoperative quadriceps and hamstring strength than those in the nonrecovery group (Table 1).

**Table 1:** Comparison between the recovery and nonrecovery groups

	All patients (*N* = 646)	The nonrecovery group (*n* = 232)	The recovery group (*n* = 414)	*p* value
Age, year	24.9 ± 10.5	28.1 ± 11.4	23.1 ± 9.6	< 0.001*
Sex, Female	322 (49.8)	126 (54.3)	196 (47.3)	n.s
Body mass index, kg/m^2^	22.5 ± 3.2	23.0 ± 3.6	22.2 ± 3.0	0.003*
Preinjury sport level				< 0.001*
Athlete	336 (52.0)	91 (39.2)	245 (59.2)	
Recreation	242 (37.5)	104 (44.8)	138 (33.3)	
No sport	68 (10.5)	37 (15.9)	31 (7.5)	
Waiting time, months	14.6 ± 43.6	21.2 ± 58.6	10.9 ± 32.0	n.s
Surgery technique, Double bundle	496 (76.8)	179 (77.2)	317 (76.6)	n.s
Medial meniscus injury, yes	204 (31.6)	84 (36.2)	120 (29.0)	n.s
Lateral meniscus injury, yes	166 (25.7)	65 (28.0)	101 (24.4)	n.s
Cartilage injury, yes	67 (10.4)	28 (12.1)	39 (9.4)	n.s
Preoperative knee function				
LSI of quadriceps strength, %	67.7 ± 22.8	57.9 ± 21.3	71.6 ± 22.1	< 0.001*
LSI of hamstring strength, %	77.6 ± 24.0	73.1 ± 26.0	80.1 ± 22.5	< 0.001*
Anterior tibial translation, mm	5.3 ± 2.7	5.4 ± 2.8	5.2 ± 2.6	n.s
Postoperative Tegner activity scale score^a^	6 [3–10]	5 [3–10]	7 [3–10]	< 0.001

Values are present as average ± standard deviation, median [minimum – maximum], and *n* (%)

*LSI* Limb symmetry index

^a^Missing data for 31 patients

*p < 0.05

**Fig. 2:**
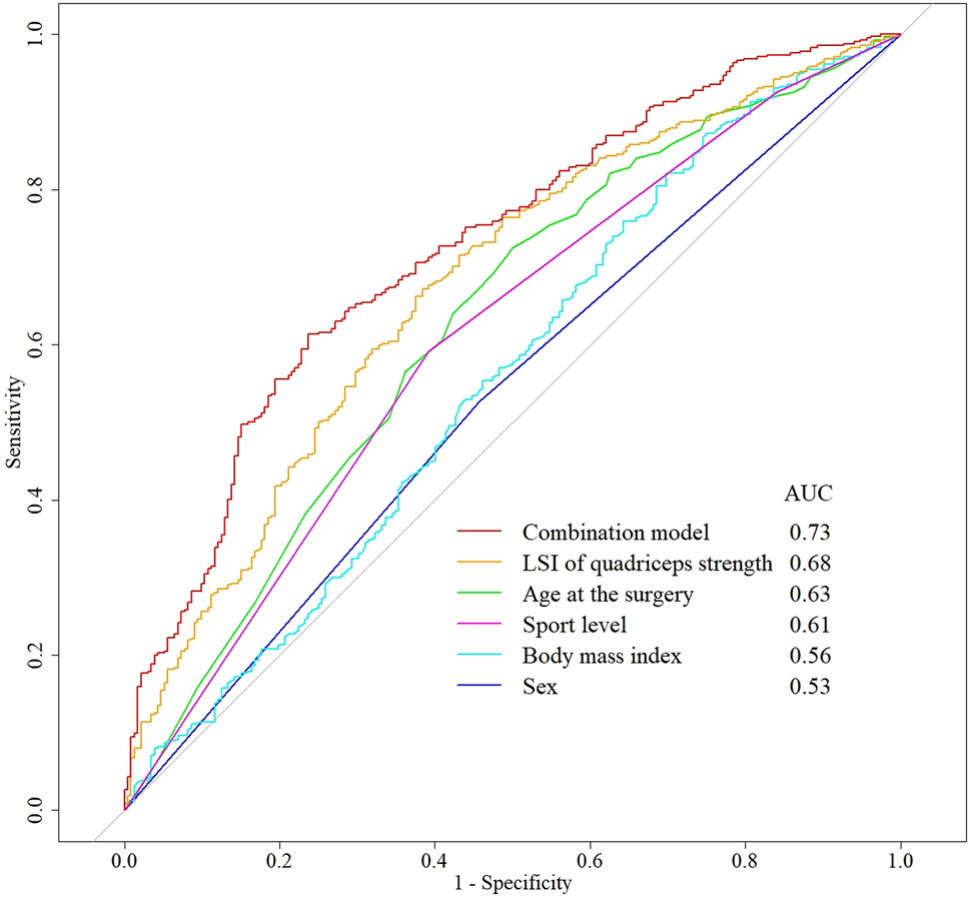
Receiver operating characteristic curves of each predictor. The combination model consisting of age, sex, body mass index, preinjury sport level, and LSI of quadriceps strength showed the highest AUC compared with that of each predictor. *AUC* Area under the curve, *LSI* Limb symmetry index

The results of the multivariate logistic regression analysis are shown in Table 2. Based on the results, a model assessing the combination of age at the time of surgery, sex, BMI, preinjury sport level, and LSI of preoperative quadriceps strength was created. Their predictive capabilities are listed in Table 3. The model assessing the predictive ability of a combination of factors had a significantly higher AUC than the model assessing the predictive ability of each independent predictor (Table 4 and Fig. 2).

**Table 2:** Result of the multivariate logistic regression analysis

Predictor	OR	95% CI	*p* value
Age	0.97	0.95–0.99	0.009*
Sex			
Male	Ref	–	–
Female	0.68	0.47–0.99	0.049*
Body mass index	0.93	0.88–0.99	0.022*
Preinjury sport level			
Athlete	Ref	–	–
Recreation	0.75	0.47–1.17	n.s
No sport	0.52	0.27–0.99	0.048*
Waiting time	0.99	0.99–1.01	n.s
Surgery technique			
Single bundle	Ref	–	–
Double bundle	1.00	0.67–1.52	n.s
Medial meniscus injury			
No	Ref	–	–
Yes	1.02	0.68–1.52	n.s
Lateral meniscus injury			
No	Ref	–	–
Yes	1.06	0.70–1.61	n.s
Cartilage injury			
No	Ref	–	–
Yes	1.18	0.64–2.14	n.s
LSI of quadriceps strength	1.04	1.03–1.05*	< 0.001*
LSI of hamstring strength	0.99	0.98–1.00	n.s
Anterior tibial translation	0.97	0.91–1.04	n.s

*DB* double bundle, *LSI* limb symmetry index, *OR* odds ratio, *CI* confidence interval

*p < 0.05

**Table 3:** Predictive ability of each independent predictor and the combination model for quadriceps strength recovery

	Predictor	Accuracy	Sensitivity	Specificity	PPV	NPV	Cut-off value
A	Age	0.64	0.72	0.50	0.72	0.50	22.5 years
B	Sex	0.53	0.53	0.54	0.67	0.39	–
C	Body mass index	0.63	0.82	0.30	0.68	0.49	24.3 kg/m^2^
D	Preinjury sport level	0.60	0.59	0.61	0.73	0.45	–
E	LSI of quadriceps strength	0.65	0.67	0.62	0.76	0.51	63.3%
F	The combination model	0.67	0.61	0.76	0.82	0.53	–

*PPV* positive predictive value, *NPV* negative predictive value, *LSI* limb symmetry index

**Table 4:** Comparison of C-statistics for each independent predictor and the combination model for quadriceps strength recovery

	Predictor	AUC	vs. B	vs. C	vs. D	vs. E	vs. F
A	Age	0.63 (0.58–0.67)	0.005	0.013	n.s	n.s	< 0.001
B	Sex	0.53 (0.49–0.58)	–	n.s	0.016	< 0.001	< 0.001
C	Body mass index	0.56 (0.51–0.61)	–	–	n.s	< 0.001	< 0.001
D	Preinjury sport level	0.61 (0.57–0.65)	–	–	–	0.029	< 0.001
E	LSI of quadriceps strength	0.68 (0.63–0.72)	–	–	–	–	0.002
F	The combination model	0.73 (0.69–0.77)	–	–	–	–	–

The combination model consisted of age, sex, body mass index, preinjury sport level, and LSI of quadriceps strength

*AUC* area under the curve, *LSI* limb symmetry index

**Fig. 3:**
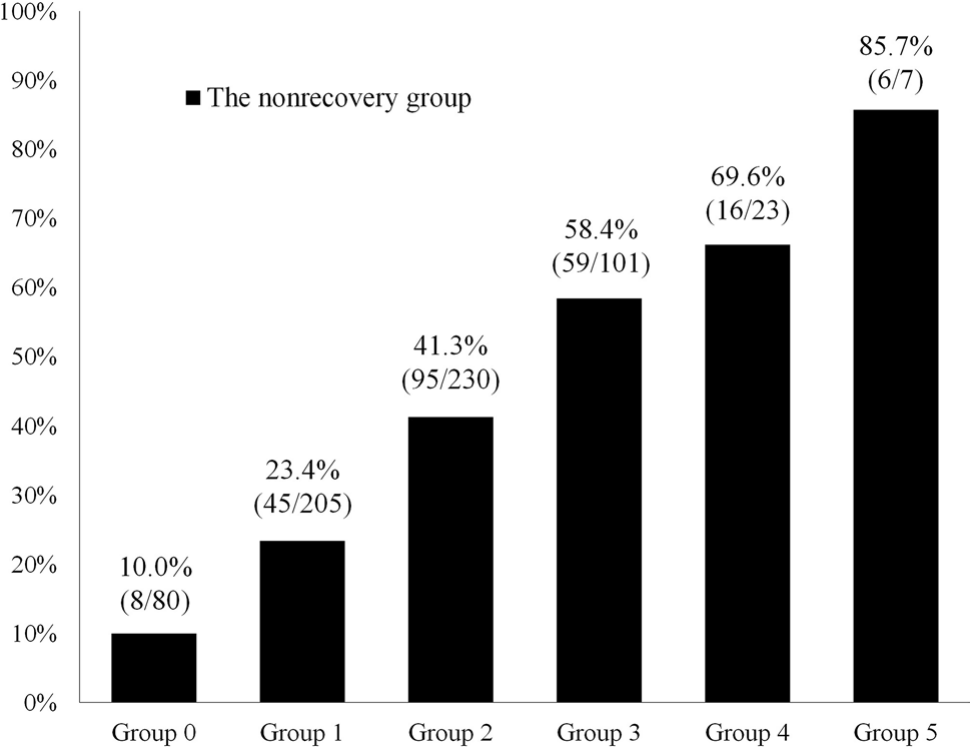
Proportion of quadriceps strength recovery groups according to the number of cut-off values met. The cut-off values were as follows: age > 22.5 years, sex; female, body mass index > 24.3 kg/m^2^, preinjury sport level; no sport, and limb symmetry index of preoperative quadriceps strength < 63.3%. The percentage of patients included in the nonrecovery group significantly increased as the number of cut-off values met increased

The NRI and IDI showed that the model assessing the predictive ability of a combination of factors had better reclassification and discrimination capabilities that of each independent predictor (Table 5). Furthermore, patients were classified into six groups (0–5) according to the number of the following five cut-off values met. The percentage of patients included in the nonrecovery group significantly increased with the number of cut-off values met (*p* < 0.001) (Fig. 3).

**Table 5:** Comparison of discrimination for the combination model and each independent predictor for quadriceps strength recovery

The combination model	NRI (95% CI)	*p* value	IDI (95% CI)	*p* value
vs. Age	0.606 (0.453–0.759)	< 0.001	0.096 (0.072–0.120)	< 0.001
vs. Sex	0.653 (0.500–0.805)	< 0.001	0.142 (0.113–0.170)	< 0.001
vs. Body mass index	0.671 (0.519–0.822)	< 0.001	0.131 (0.103–0.158)	< 0.001
vs. Preinjury sport level	0.544 (0.389–0.699)	< 0.001	0.105 (0.080–0.129)	< 0.001
vs. LSI of quadriceps strength	0.492 (0.336–0.648)	< 0.001	0.060 (0.040–0.080)	< 0.001

The combination model consisted of age, sex, body mass index, preinjury sport level, and LSI of quadriceps strength

*NRI* net reclassification improvement, *IDI* integrated discrimination improvement, *LSI* limb symmetry index

## Discussion

The most important finding of the present study was that the predictive ability of the model combining independent predictors for quadriceps strength recovery at one year after ACL reconstruction was better than that of each independent predictor. This result suggests that multiple factors, including patient demographics and preoperative quadriceps strength, should be considered when predicting quadriceps strength recovery after ACL reconstruction.

This study described the independent predictors of quadriceps strength recovery one year after ACL reconstruction. Previous studies have reported that age, sex, BMI, preinjury activity level, and preoperative quadriceps strength are associated with postoperative quadriceps strength recovery after ACL reconstruction, and our results are consistent with these findings. However, only a few studies have examined the predictive ability of these predictors. The present study showed that the preoperative LSI of quadriceps strength had the highest AUC value among the independent predictors alone, followed by age, preinjury sport level, BMI, and sex. These results suggest that preoperative quadriceps strength and age may be predominant factors for predicting postoperative quadriceps strength recovery. However, the AUC of each predictor was lower than that of the combination of predictors. Ueda et al. [29] reported the predictive ability of the preoperative LSI of quadriceps strength for the postoperative LSI of quadriceps strength recovery six months after ACL reconstruction in athletes and indicated a low AUC value. The results of the current study support previous research, suggesting that the predictive ability of preoperative independent predictors alone may not be sufficient to predict postoperative quadriceps strength recovery.

The present study revealed that the combination of patient demographics and preoperative quadriceps strength improved the prediction, reclassification, and discrimination abilities of quadriceps strength recovery after ACL reconstruction. This is the first study to show that a combination of independent predictors improves the predictive ability of quadriceps strength recovery after ACL reconstruction. In addition, the proportion of patients in the nonrecovery group significantly increased with the number of cut-off values met. This study suggests that patient characteristics and preoperative knee function should be comprehensively evaluated to predict quadriceps strength recovery after ACL reconstruction. Patients who are predicted to have poor quadriceps strength recovery should participate in appropriate preoperative and postoperative rehabilitation programs according to their predictors. In contrast, the predictive ability of the combination of factors was significantly better; moreover, the values indicating predictive capability, such as sensitivity, specificity, and accuracy, were not clinically significant. Postoperative knee extension restriction [12] and anterior knee pain [15, 29] are reported to affect the postoperative recovery of quadriceps strength. Therefore, further studies are needed to determine whether a prediction model with sufficient clinical predictive ability can be created by adding immediate postoperative knee function as a predictor or by using a different statistical model.

This study had some limitations. First, this study was conducted in a single center, and many patients were excluded because of missing data. Therefore, the results should externally validated in multicenter studies and other centers for external validity. Second, it is unclear why the combination of independent predictors had such a superior predictive ability. Third, the present study sets an LSI of quadriceps strength of 80% as the cutoff value to indicate poor quadriceps strength recovery one year after surgery. Nevertheless, it is unclear whether the predictive ability would change if other values, such as 85% or 90%, were set as the cutoff value. Fourth, two isokinetic dynamometers were used to evaluate quadriceps and hamstring strength due to mechanical failure. Although we conducted a test for agreement of values between the two instruments, the results should be interpreted with caution because of the small sample of subjects. Fifth, although the knee muscle strength test was performed within two weeks before surgery in most of the patients, the timing of the measurements varied among the patients. Therefore, knee muscle strength at the time of surgery may have changed from that at the time of measurement, thus affecting the results. Finally, the same surgical treatment and postoperative time-based rehabilitation protocol were used, although some of these interventions may have changed slightly over the long study periods. Additionally, the motivation of the patient, adherence to rehabilitation, and time to return to sports were not considered. These limitations may explain the low predictive ability of this study. Despite these limitations, this study could be useful in the preoperative phase to identify patients who may have poor quadriceps strength after ACL reconstruction.

## Conclusion

This study showed that the predictive ability of the combination of independent predictors for quadriceps strength recovery at one year after ACL reconstruction was superior to that of the independent predictors alone. Multiple factors, including patient characteristics and preoperative quadriceps strength, should be considered when predicting quadriceps strength recovery after ACL reconstruction.

## Data Availability

Data supporting the findings of this study are available from the corresponding author on reasonable request.

## Abbreviations

ACL: Anterior cruciate ligament
AUC: Area under the curve
BMI: Body mass index
BTB: Bone-patellar tendon-bone
DB: Double bundle
IDI: Integrated discrimination improvement
LSI: Limb symmetry index
NRI: Net reclassification improvement
ROC: Receiver operating characteristic
ROM: Range of motion
SB: Single bundle
